# The Diagnosis of Protein Energy Wasting in Chronic Peritoneal Dialysis Patients Is Influenced by the Method of Calculating Muscle Mass. A Prospective, Multicenter Study

**DOI:** 10.3389/fmed.2021.702749

**Published:** 2021-08-25

**Authors:** Cristina Techy Roth-Stefanski, Naiane Rodrigues de Almeida, Gilson Biagini, Natália K. Scatone, Fabiana B. Nerbass, Thyago Proença de Moraes

**Affiliations:** ^1^Pontificia Univerdidade Catolica do Parana, Post Graduate Program in Health and Biological Sciences, Curitiba, Brazil; ^2^Santa Casa de Misericórdia de Curitiba, Curitiba, Brazil; ^3^Instituto Do Rim, Curitiba, Brazil; ^4^Fundação Pró-Rim, Joinville, Brazil

**Keywords:** kidney nutrition, malnutrition, PEW, ESKD, peritoneal dialysis

## Abstract

**Objective:** To analyze the concordance and agreement between bioimpedance spectroscopy (BIS) and anthropometry for the diagnosis of protein energy wasting (PEW) in chronic peritoneal dialysis patients.

**Methods:** Prospective, multi-center, observational study using multifrequency bioimpedance device (*Body Composition Monitor -BCM*^®^*- Fresenius Medical Care*) and anthropometry for the diagnosis of PEW as recommended by the International Society of Renal Nutrition and Metabolism (ISRNM). Cohen's kappa was the main test used to analyze concordance and a Bland-Altmann curve was built to evaluate the agreement between both methods.

**Results:** We included 137 patients from three PD clinics. The mean age of the study population was 57.7 ± 14.9, 47.8% had diabetes, and 52.2% were male. We calculated the scores for PEW diagnosis at 3 and 6 months after the first collection (T3 and T6) and on average 40% of the study population were diagnosed with PEW. The concordance in the diagnosis of PEW was only moderate between anthropometry and BIS at both T3 and T6. The main factor responsible for our results was a low to moderate correlation for muscle mass in kilograms, with an r-squared (R2) of 0.35. The agreement was poor, with a difference of more than 10 kg of muscle mass on average and with more than a quarter of all cases beyond the limits of agreements.

**Conclusion:** Current diagnosis of PEW may differ depending on the tools used to measure muscle mass in peritoneal dialysis patients.

## Introduction

Protein energy wasting (PEW) is a common condition in patients with chronic kidney disease (CKD). Its incidence and severity increase as the renal disease progresses to kidney failure, with a peak observed in dialysis patients (1, 2). Depending on the modality of choice, new risk factors for PEW are introduced. In peritoneal dialysis (PD), exposure to glucose as an osmotic agent may lead to an absorption of up to 300 g glucose per day, depending on the patient's membrane profile and the prescription of hypertonic solutions. Such glucose load has a direct impact on the patient's appetite, reducing the daily intake of proteins and other nutrients (3, 4). In addition, patients have a daily loss of protein through the peritoneal membrane, which in some cases may reach 10 g, what can contribute to the deterioration of the nutritional status (5, 6).

Early diagnosis of PEW is of particular importance because advanced states of malnutrition and inflammation may be difficult to reverse and also because these patients are more likely to have a poor quality of life and a higher risk of death from any cause (2, 7). Standardization of the diagnosis of PEW occurred when the International Society of Renal Nutrition and Metabolism (ISRNM) established these criteria for PEW in 2008 (7). This criterion includes serum biomarkers, data on dietary intake and the traditional nutritional physical examination. The latter includes the calculation of muscle mass loss by means of repeated measures of the mid-arm muscle circumference area between a pre-established period of time. This procedure is part of a time-consuming, operator-dependent physical examination and, consequently, prone to significant variance.

The introduction of bioimpedance spectroscopy (BIS) into clinical practice in nephrology has improved the care of dialysis patients in different forms (8). Of our interest, BIS quickly allows the automatic measurement of lean body mass (LBM). It is important to make a distinction before further discussion, LBM is the non-mineral component of free fat mass that is measured with traditional bioimpedance technologies using two compartment models. BIS-measured LBM has already been described as an important predictor of survival in adults treated with chronic hemodialysis (9, 10). Given the potential variability in muscle mass quantification between BIS and anthropometry, and that this parameter is important for the diagnosis of PEW, we designed a study to analyze the concordance between BIS and anthropometry for the diagnosis of PEW. Our hypothesis was that the concordance between the methods differs considerably.

## Methods

This is a prospective, multi-center, observational study designed to examine the concordance between BIS and anthropometry for the diagnosis of PEW. Secondary objectives of the study were to compare the concordance between the methods for measuring muscle mass and the scores for diagnosing PEW.

### Patients and Settings

PD patients were recruited from three centers in Southern Brazil between June 2018 and January 2020. Only patients older than 18 years old, undergoing PD for >3 months were included. Exclusion criteria were pregnancy; body mass index (BMI) >35 kg/m^2^; major limb amputations; disability (need for wheelchair); active cancer diagnosis; diagnosis of severe liver failure; patients with pacemaker, abuse of alcohol, or illegal drugs history.

Demographic data were collected at baseline from patients' medical records (comorbidities, dialysis vintage, previous dialysis therapies and cause of CKD) whilst biochemical data (creatinine, albumin, phosphorus, and hemoglobin) were recorded quarterly. Participants were also inquired about the use of dietary supplements and physical activity. The ethics committee of the Pontificia Universidade Católica do Paraná approved the research protocol under the number 4.086.745, and all participants provided a written informed consent form.

### Study Size

The sample size calculation was based on a pilot study with 39 patients. Patients were classified into two groups according to their PEW score (1–2 and 3–4) using the two methods chosen for this study for the diagnosis of PEW (classical and BIS). We designed the study for a power of 0.8 and established the significance level of alpha at 0.05. We estimated that 110 patients would be necessary to identify a 15% difference in the concordance between methods.

### Body Composition

#### Anthropometry

Nutritional parameters measured included: dry body weight (patients were weighed with light clothing and no shoes on a platform manual scale balance), height, body mass index (BMI), mid arm circumference (MAC), and skinfold measurements. These were taken at four sites (biceps, triceps, subscapular and suprailiac) on the opposite side of the vascular access (if the patient had the vascular access) using the Cescorf skinfold caliper (Cescorf Scientific, Porto Alegre, RS, Brazil). The mean of three measurements for each skinfold was taken. The sum of skinfold thicknesses at four sites allowed obtaining the body fat percentage using the table published by Durnin and Womersley (11).

Muscle mass was obtained by subtracting total body fat (in kilograms) and total corporal water (estimated by Watson formula) from total body weight. The midarm muscle circumference (MAMC), was assessed by standard methods and classified according to percentile distribution tables adapted by Frisancho (12).

#### Bioimpedance

The estimated parameters of the body composition monitor were overhydration (OH), lean tissue mass (LTM), fat tissue mass (FTM), and relative fat in percentage, using multifrequency bioimpedance device (BCM®). The technique is performed by attaching electrodes to the patient's non-fistula forearm and ipsilateral ankle, with the patient in a supine position. The BCM then applies an imperceptible electrical discharge that measures body resistance and reactance to electric current and uses it to provide information on several body composition parameters (13). We followed all the manufacturer's recommendations.

### Diagnosis of PEW

The diagnosis of PEW was established as recommended by ISRNM (7). Four distinct categories are taken into account for the diagnosis: (1) biochemical parameters, (2) low body weight, reduced body fat or weight loss, (3) decreased muscle mass, and (4) low protein or energy intake. The Supplementary Table 1 provides additional details on the parameters used in our study.

Clinical, biochemical, nutritional and body composition measurements were taken to assess the patients' nutritional status at baseline (T0) and 3 (T3) and 6 (T6) months after.

The criteria used to calculate the score included biochemical data (serum albumin); body mass (low body weight, reduced total body fat, or weight loss); muscle mass (decreased muscle mass and reduced mid-arm muscle circumference area) and dietary intake (see Supplementary Table 1). At least one test in each of the four categories must be satisfied for the diagnosis of kidney disease-related PEW.

The diagnosis of PEW was made with the data obtained through classical anthropometry and with the body composition data obtained through BCM at T3 and T6. The weight used in the calculation of BMI and weight loss when making the diagnosis of PEW through BCM, was the measured weight value subtracted from the OH value found by bioimpedance.

### Statistical Analysis

Continuous variables were expressed as mean ± SD or median and interquartile range, while categorical variables (e.g., gender, race, primary renal disease, presence of comorbid conditions, initial therapy, and current PD modality) were expressed as frequencies or percentages. The χ^2^, *t*-test, or Wilcoxon were used, as appropriate, to compare demographic and clinical characteristics at baseline. For the concordance between methods, we used primarily the Cohen's kappa, and for exploratory reasons, we also reported Fleiss Kappa, Gwetá AC, Krippendorff's alpha, and Brennan & Predifer agreement. To explore the association between muscle mass in both methods we used Passin and Bablock regression and for concordance, we also performed Lins coefficient. In addition, we made a graph of the correlation among the muscle mass values between both methods by adding a line of the estimated values using a fractional polynomial which in turn was calculated using the regression model described by Roston and Altman in 1994. Finally, we also made a Bland-Altman curve to evaluate the agreement between methods. Statistical significance was set at the level of *p* < 0.05. All analyses were performed using STATA 14.0.

## Results

**Figure 1:**
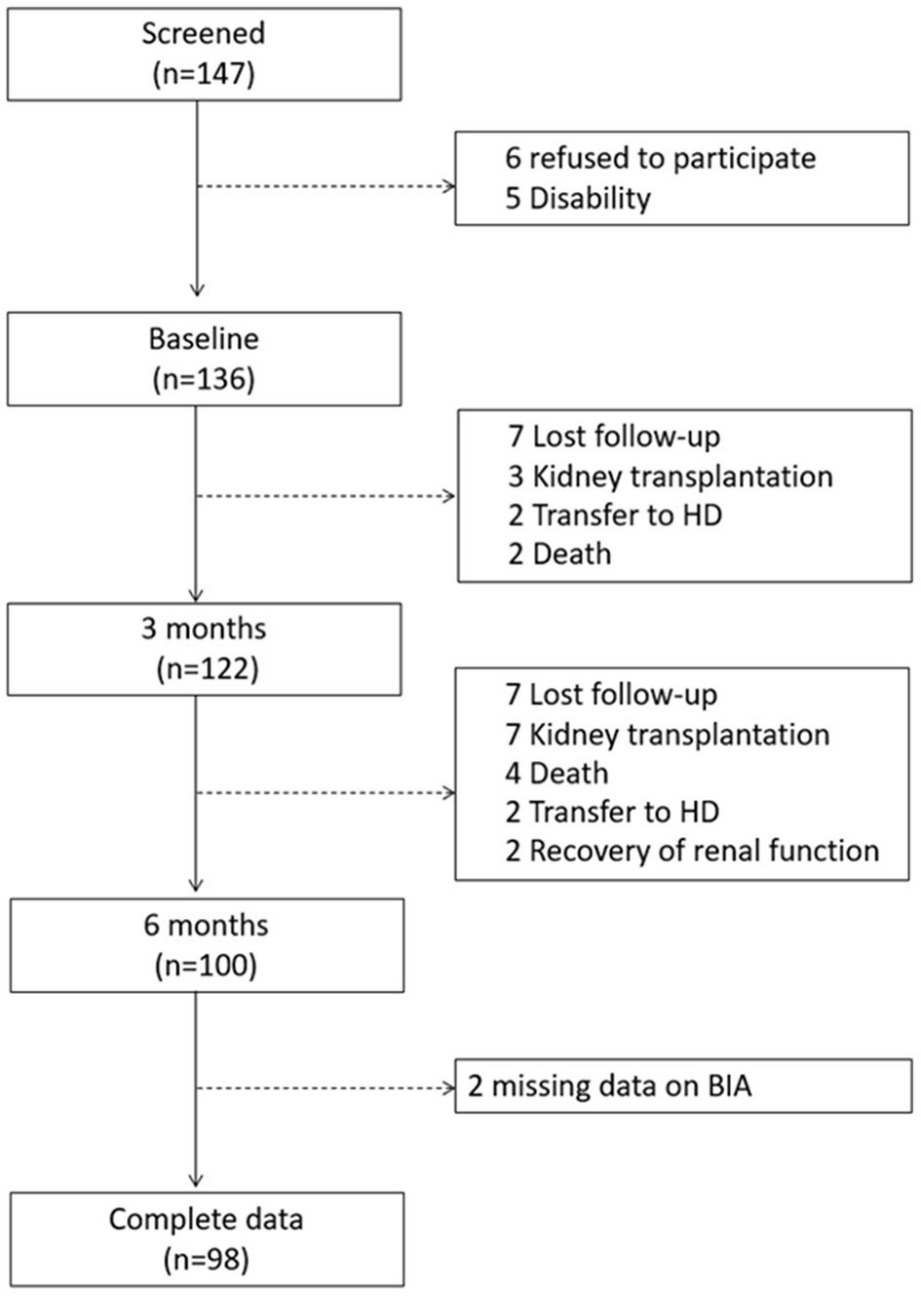
Study flowchart.

We included 137 patients from 3 PD clinics located in Southern Brazil. The mean age of the study population was 57.7 ± 14.9, 47.8% had diabetes, and 52.2% were male. More details on demographics can be found in Table 1. Only four patients had a diagnosed episode of peritonitis, there were 24 admissions and four deaths during the study. Only two patients received a renal transplantation (Figure 1).

**Table 1:** General characteristics of the study population.

**Clinical**	
Age (years)	57.7 ± 14.9
Body mass index (kg/m^2^)	26.9 ± 5.3
Overhydration (L)	1.5 (IQR 0 – 5.3)
**Demographic**	
Diabetes (yes)	47.8% (*n* = 65)
CKD etiology	
*Diabetes*	26.5% (*n* = 36)
*Glomerulonephritis*	19.8% (*n* = 27)
*Hypertension*	19.1% (*n* = 26)
*Post-renal*	2.2% (*n* = 03)
*Unknown*	16.9% (*n* = 23)
*Others*	15.4% (*n* = 21)
Gender (Male)	52.2% (*n* = 71)
Hypertension (yes)	77.9% (*n* = 106)
Oral supplement (yes)	5.9% (*n* = 08)
Previous hemodialysis (yes)	36.8% (*n* = 50)
Race (White)	79.4% (*n* = 26)
Routine exercise (yes)	21.3% (*n* = 29)
**Laboratorial**	
Albumin (g/dl)	3.7 ± 0.7

*CKD, Chronic kidney disease; IQR, Interquartile range*.

The nutritional status of the study population showed 40% of them with protein energy wasting based on the ISRN criteria. All nutritional parameters calculated at baseline, using anthropometrics and BIS, and stratified by gender, are described in Table 2.

**Table 2:** Nutritional parameters at baseline.

	**Overall**	**Male**	**Female**
**Anthropometrics**			
Arm muscle circumference (mm)	240.9 ± 39.9	248.4 ± 40.2	232.7 ± 38.3
Biceps skinfold (mm)	14.0 ± 8.7	10.8 ± 6.0	17.5 ± 9.8
Body fat (kg)	24.5 ± 9.1	22.4 ± 8.5	26.7 ± 9.3
Body mass index (kg/m^2^)	26.9 ± 5.3	26.4 ± 4.5	27.4 ± 15.7
Muscle mass (kg)	48.2 ± 9.3	54.2 ± 7.2	41.7 ± 6.6
Mid-upper arm circumference (cm)	29.7 ± 4.9	28.9 ± 4.3	30.5 ± 5.5
Subcapsular skinfold (mm)	23.4 ± 10.5	21.8 ± 9.4	25.0 ± 11.5
Supra-iliac skinfold (mm)	21.3 ± 10.1	20.1 ± 9.5	22.5 ± 10.7
Triceps skinfold (mm)	17.8 ± 8.7	13.1 ± 6.2	22.9 ± 8.2
*Protein Energy Wasting (yes)*	35%	30%	40%
**PEW score**			
0–1	35%	30%	40%
2	48%	53%	43%
3	11%	11%	11%
4	6%	6%	6%
**Bioimpedance Spectroscopy (BIS)**			
Fat tissue mass (kg)	25.9 ± 10.8	24.4 ± 10.0	27.5 ± 11.3
Lean tissue mass (kg)	35.7 ± 10.8	41.4 ± 10.7	29.5 ± 6.6
*Protein Energy Wasting (yes)*	40%	46%	35%
**PEW score**			
0–1	40%	46%	35%
2	37%	31%	43%
3	15%	13%	17%
4	7%	10%	4%

*PEW, Protein Energy Wasting*.

**Figure 2:**
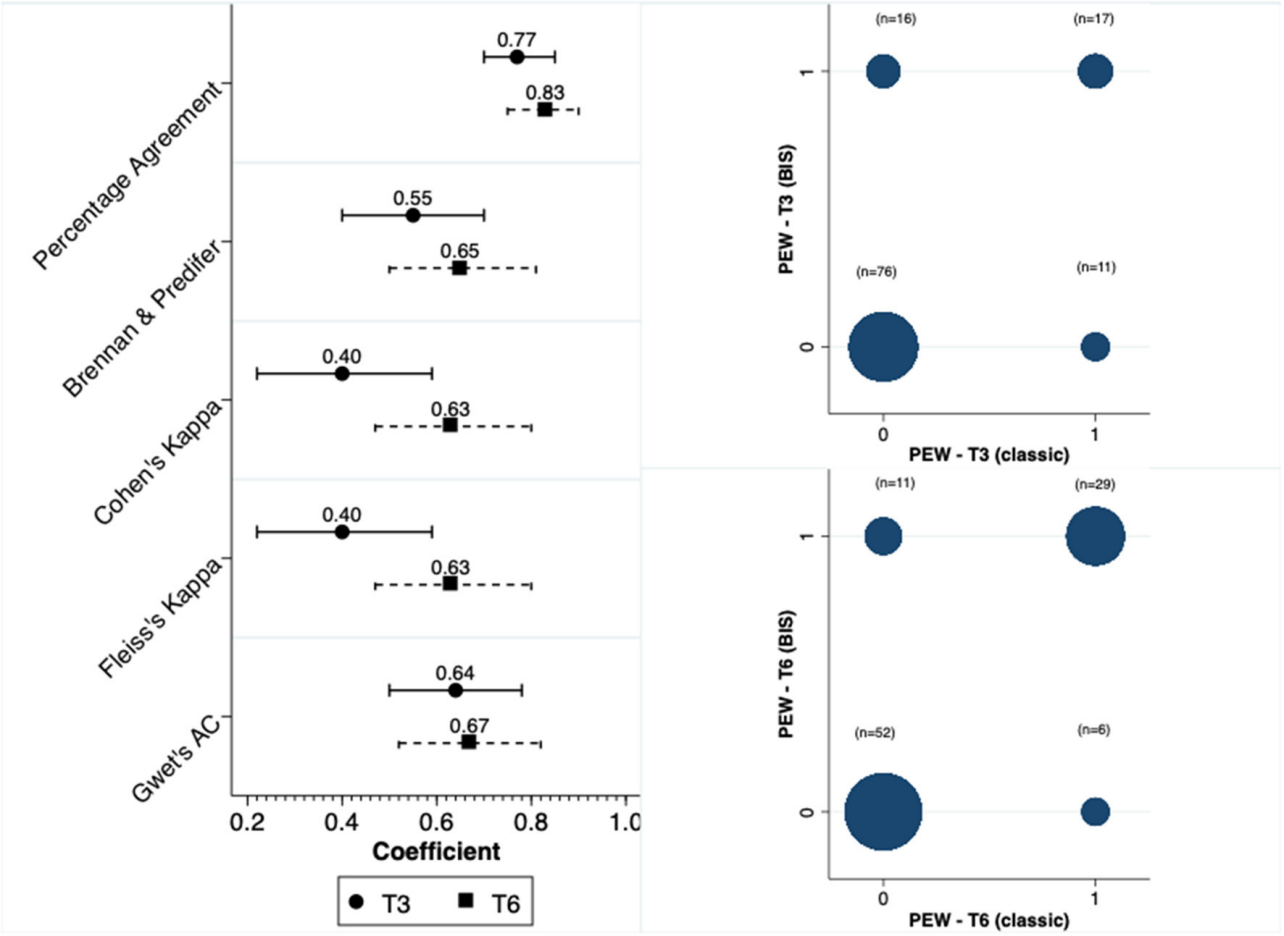
Concordance and distribution of PEW diagnosis.

We calculated the scores for the diagnosis of PEW at two distinct moments, at 3 and 6 months. Concordance in the diagnosis of PEW was moderate between anthropometry and BIS at both T3 and T6. The concordance was higher at T6 compared to T3. Figure 2 depicts this concordance and the distribution of diagnosis in both moments for the two methods.

**Figure 3:**
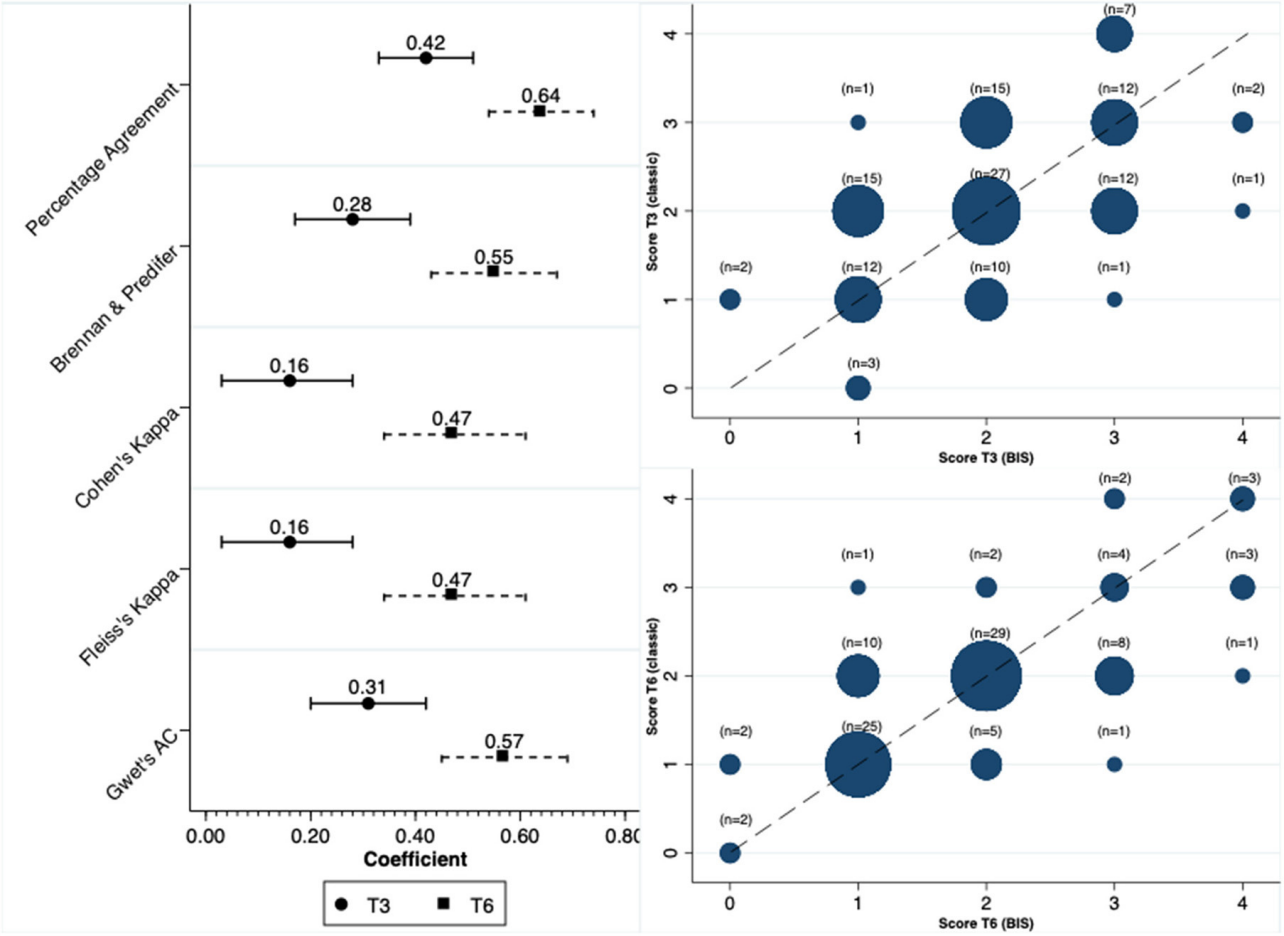
Concordance between the score used for diagnosing PEW.

In contrast to the concordance observed for the diagnosis of PEW, the concordance was significantly reduced when we analyzed the total score. In line with our findings for PEW diagnosis, the scores at the time of 6 months had a better concordance compared to the 3-month results (Figure 3). In terms of the parameters that were constant between methods, and respectively at T3 and T6, the percentage of patients with serum albumin level <3.8 mg/dl was 56 and 58%, with BMI < 23 kg/m^2^ was 18 and 19% and with a low dietary intake 20.5 and 21%.

**Figure 4:**
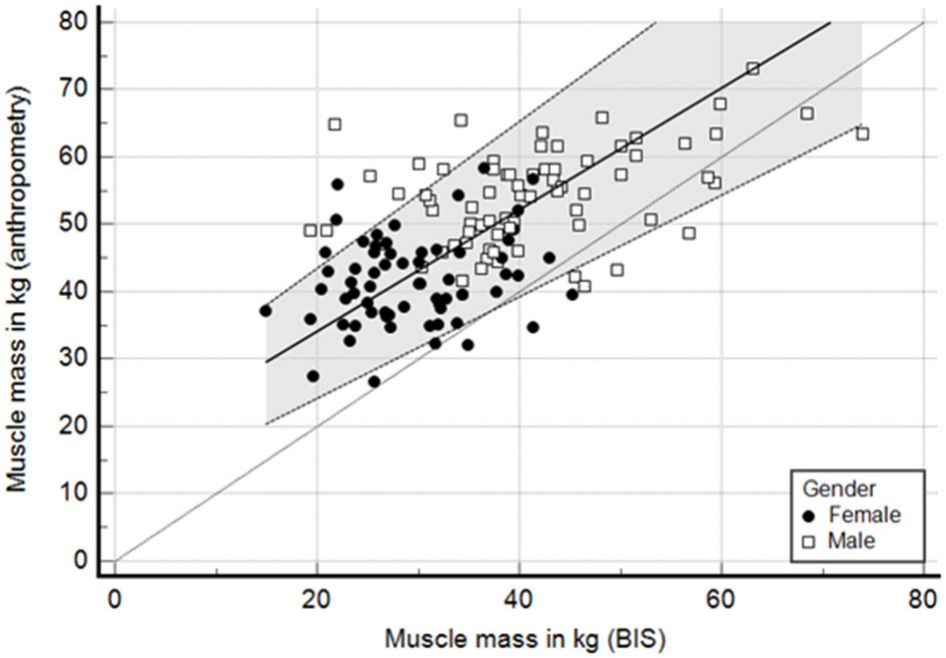
Passing and bablok regression—correlation of muscle mass (kg) between anthropometry and BIS. Lins concordance 0.33.

To understand the lack of concordance between both methods, we analyzed the correlation and agreement for muscle and fat mass. There was a low to moderate correlation for muscle mass in kilograms, with *R*^2^ of 0.35 (Figure 4). At the bottom of Figure 4 we show the distribution of delta values for muscle mass that contribute to the understanding of the larger variability between methods. In addition, we explored the same correlation but in the subgroup of patients with BMI below and above 30 kg/m^2^. The *R*^2^ for patients with BMI < 30 kg/m^2^ was 0.32 and for those with BMI ≥ 30 kg/m^2^ 0.59 (Supplementary Figures 2, 3). In contrast to muscle mass, the correlation for fat mass was much better with an *R*^2^ of 0.63.

**Figure 5:**
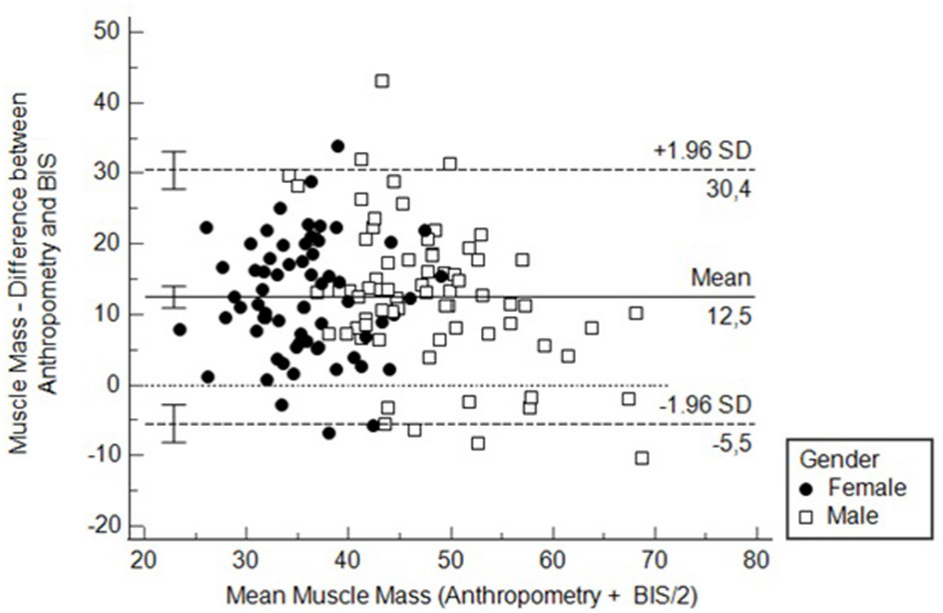
Bland-Altmann curve to assess the agreement between BIS and anthropometry for measuring muscle mass. MMA, Muscle mass measured by anthropometry; MMB, Muscle mass measured by bioimpedance spectroscopy.

Finally, we assessed the agreement with a Bland-Altmann curve. The agreement was poor, with a difference of more than 10 kg of muscle mass on average and with more than a quarter of all cases beyond limits of agreements (Figure 5). In contrast, the agreement for fat mass was apparently better, with a difference close to 1 kg. However, the variability was high with 30% of cases beyond the limits of agreement (Supplementary Figure 5).

## Discussion

In this prospective, multicenter, cohort study, we observed a poor agreement for the diagnosis of PEW between anthropometry and BIS in PD patients. The main differences found were due to a lack of agreement in the quantification of the participants' muscle mass in PD patients. The muscle mass calculated using anthropometry was significantly greater compared to BIS for most patients. This lack of agreement is large, not acceptable, and can potentially impact clinical outcomes in the long term. Nevertheless, without data on patient outcomes, our study cannot endorse BIS as a reference method.

The incidence of PEW in chronic kidney disease patients (CKD) is high and increases unacceptably as the kidney function deteriorates (14). The peak in the prevalence of wasting occurs when a patient gets to dialysis, with some studies reporting signs of wasting in up to 75% of the study population (2, 7). In our cohort, the prevalence of PEW varied between 35 and 40% depending on whether we used, respectively, anthropometry or BIS for the diagnosis.

Early and correct diagnosis of PEW is of critical importance to minimize risks imposed by this condition. The ISRNM criteria for the diagnosis of PEW include repeated measurements of fat and muscle mass (7). However, great variability has been described among current body composition assessment techniques. In HD patients, the use of anthropometry to estimate fat mass performed better than bioimpedance using dual-energy X-ray absorptiometry (DEXA) as the reference method (15, 16). Nevertheless, the recently published guideline for nutrition in CKD, by the Kidney Disease Outcomes Quality Initiative (KDOQI), suggests the use of multi-frequency bioelectrical impedance to assess body composition for patients on maintenance HD with a level of evidence 2C. On the other hand, the evidence for patients on chronic PD is weaker (17).

The causes of PEW are multifactorial and include factors that promote inadequate nutrients intake or increase nutrient losses, and the inflammatory process that generally follows the loss of kidney function (18). PD patients share some characteristics that make the diagnosis of PEW challenging and, consequently, may complicate nutritional diagnosis. One known factor is lower albumin levels compared to their counterparts on HD, which is caused largely by the constant loss of protein through the peritoneal membrane (6). Another important point that deserves discussion is related to the presence of peritoneal dialysate in the peritoneal cavity when performing bioimpedance. Data suggest that the presence of peritoneal dialysate could be a potential confounder for analyzing body composition, particularly for total body water and fat mass (19, 20). However, the same does not seem to apply for LBM, which was not altered by the presence of dialysate in the peritoneal cavity, as highlighted in the KDOQI guideline. In our study, we did not ask the patient to drain the peritoneal cavity.

Our study described a low concordance and agreement between conventional anthropometry and BIS for the calculation of LBM in PD patients. This lack of agreement occurred at all times of the study. In addition, the reported differences were similar among all three PD centers included in the study. In all three centers, both anthropometry and BIS were performed by three distinct and well-trained nutritionists. More importantly, we demonstrate that such differences had a direct impact on the score used for the diagnosis of PEW. Despite the systematic differences in terms of absolute muscle mass quantification, the low agreement for the diagnosis of PEW did not seem to follow a systematic pattern. Therefore, whether these differences will reflect a better capacity to predict outcomes in favor of any method is not possible to be answered at this moment. The cohort will be followed further in the upcoming years to answer this question. Subgroup analysis stratified by gender and BMI showed no sign of heterogeneity.

Our study has some limitations, which include the lack of data on prealbumin and dual-energy x-ray absorptiometry assessment. In contrast, we have some strengths, the sample size of the study was carefully calculated and based on a pilot study, we prospectively followed the patients for the diagnosis of PEW, and also the multicenter design. Again, it is important to reinforce that, at this stage of the study, our data cannot support BIS as the reference method.

In conclusion, current diagnosis of PEW may differ depending on the tools used to measure muscle mass in PD patients. Our cohort is being followed prospectively and, in the future, we hope to understand which method is better for the predicting outcomes, including hospitalization and mortality.

## Data Availability Statement

The original contributions presented in the study are included in the article/Supplementary Material, further inquiries can be directed to the corresponding author/s.

## Ethics Statement

The studies involving human participants were reviewed and approved by Pontifical Catholic University of Paraná. The patients/participants provided their written informed consent to participate in this study.

## Author Contributions

CR-S, GB, NS, FN, and TdM: data curation. TdM: methodology, formal analysis, and funding acquisition. CR-S, NR, GB, NS, FN, and TdM: investigation. CR-S, FN, and TdM: project administration. GB, FN, and TdM: resources. TdM and FN: supervision and visualization. CR-S, NS, and FN: validation. TdM and CR-S: writing of original draft. CR-S, NR, NS, FN, and TdM: writing review and editing. All authors contributed to the article and approved the submitted version.

## Conflict of Interest

TdM is a speaker of AstraZeneca, Bayer, Baxter, Boehringer-Lilly, Siemmens and Takeda; TdM has a research grant from Baxter Brazil. The remaining authors declare that the research was conducted in the absence of any commercial or financial relationships that could be construed as a potential conflict of interest.

## Publisher's Note

All claims expressed in this article are solely those of the authors and do not necessarily represent those of their affiliated organizations, or those of the publisher, the editors and the reviewers. Any product that may be evaluated in this article, or claim that may be made by its manufacturer, is not guaranteed or endorsed by the publisher.
