# Supplementary material for: The Diagnosis of Protein Energy Wasting in Chronic Peritoneal Dialysis Patients Is Influenced by the Method of Calculating Muscle Mass. A Prospective, Multicenter Study
Source: Front Med (Lausanne). 2021 Aug 25;8:702749. doi: 10.3389/fmed.2021.702749 (PMC8424066; doi:10.3389/fmed.2021.702749)
Supplement: Supplementary file 1 [file Data_Sheet_1.pdf]

Supplementary Material

Supplementary Table 1. Diagnosis of PEW

Parameter/criteria	Availability	Score
**1) Biochemical parameters**		
• Serum albumin < 3.8g/dl	**Used**	One point added when no of the 2 criteria were met
• Serum prealbumin (transthyretin) < 30 mg/dl	**Not available**	
• Serum cholesterol < 100 mg/dl	**Used***	
**2) Body mass**		
• BMI < 23Kg/m2	**Used**	One point added when no of the 3 criteria were met
• Unintentional weight loss over time: 5% over three months or 10% over six months	**Used**	
• Total body fat percentage < 10%	**Used**	
**3) Muscle mass**		
• Muscle wasting: Reduced muscle mass 5% over three months or 10% over six months	**Used**	One point added when no of the 2 criteria were met
• Reduced mid-arm muscle circumference area (reduction > 10% in relation to the fiftieth percentile of reference population)	**Used**	
**4) Dietary intake**		
• Unintentional low DPI < 0.80 gkg−1 day−1 for at least two months for dialysis patients	**Used**	One point added when no of the 2 criteria were met
• Unintentional low dietary energy intake < 25 kcal kg−1 day−1 for at least two months	**Used**	

- At least three out of the four listed categories must be satisfied for the diagnosis of PEW.
- Measurement must be performed by a trained anthropometrist
* No one met the criteria

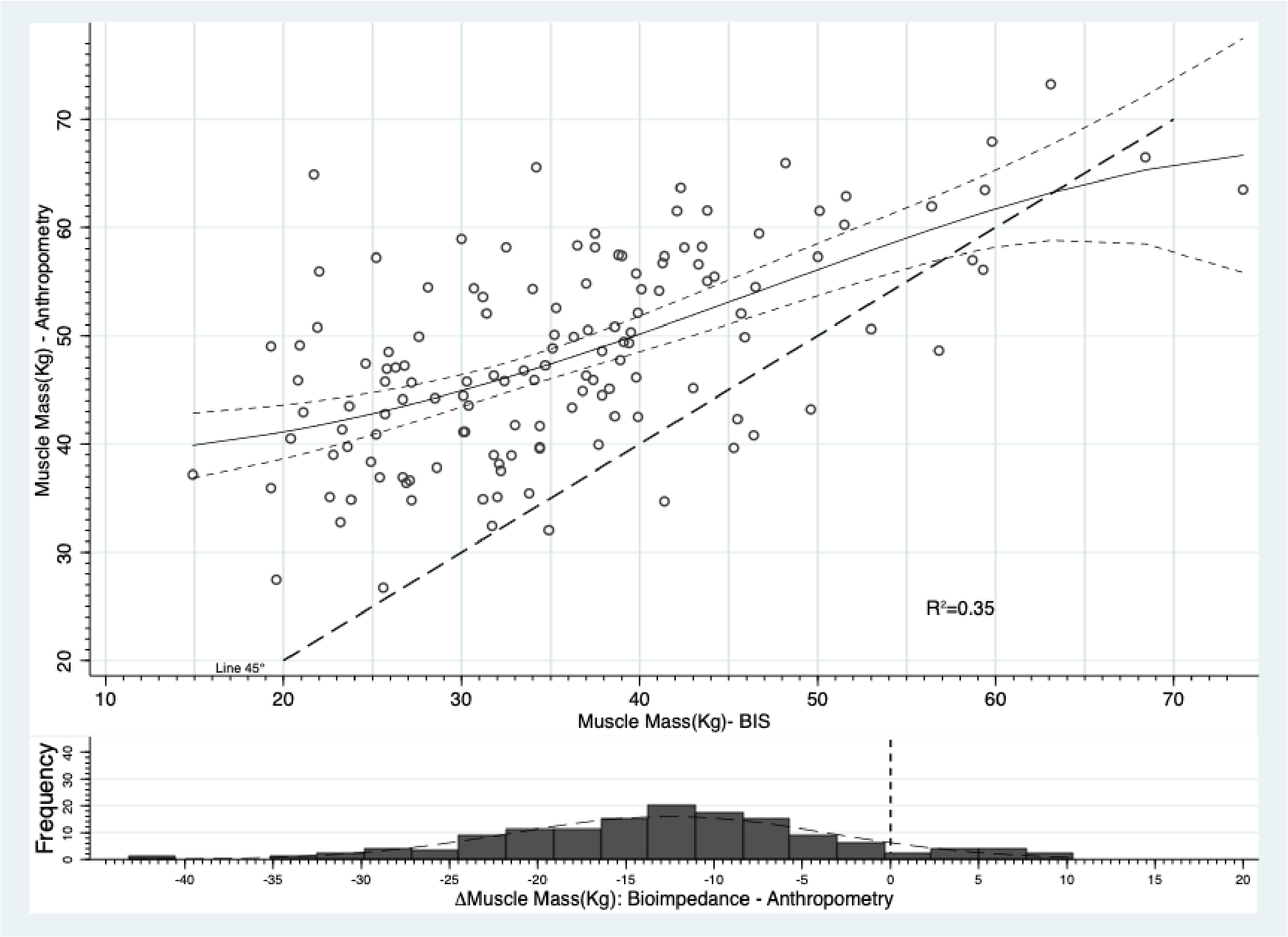

Supplementary Figure 1. Correlation of muscle mass (kg) between anthropometry and BIS

Legend: The continuous line was estimated using fractional polynomials and the gray are the CI 95%. The dashed line represents the values that would be obtained in case of a perfect correlation between methods (45° angle).

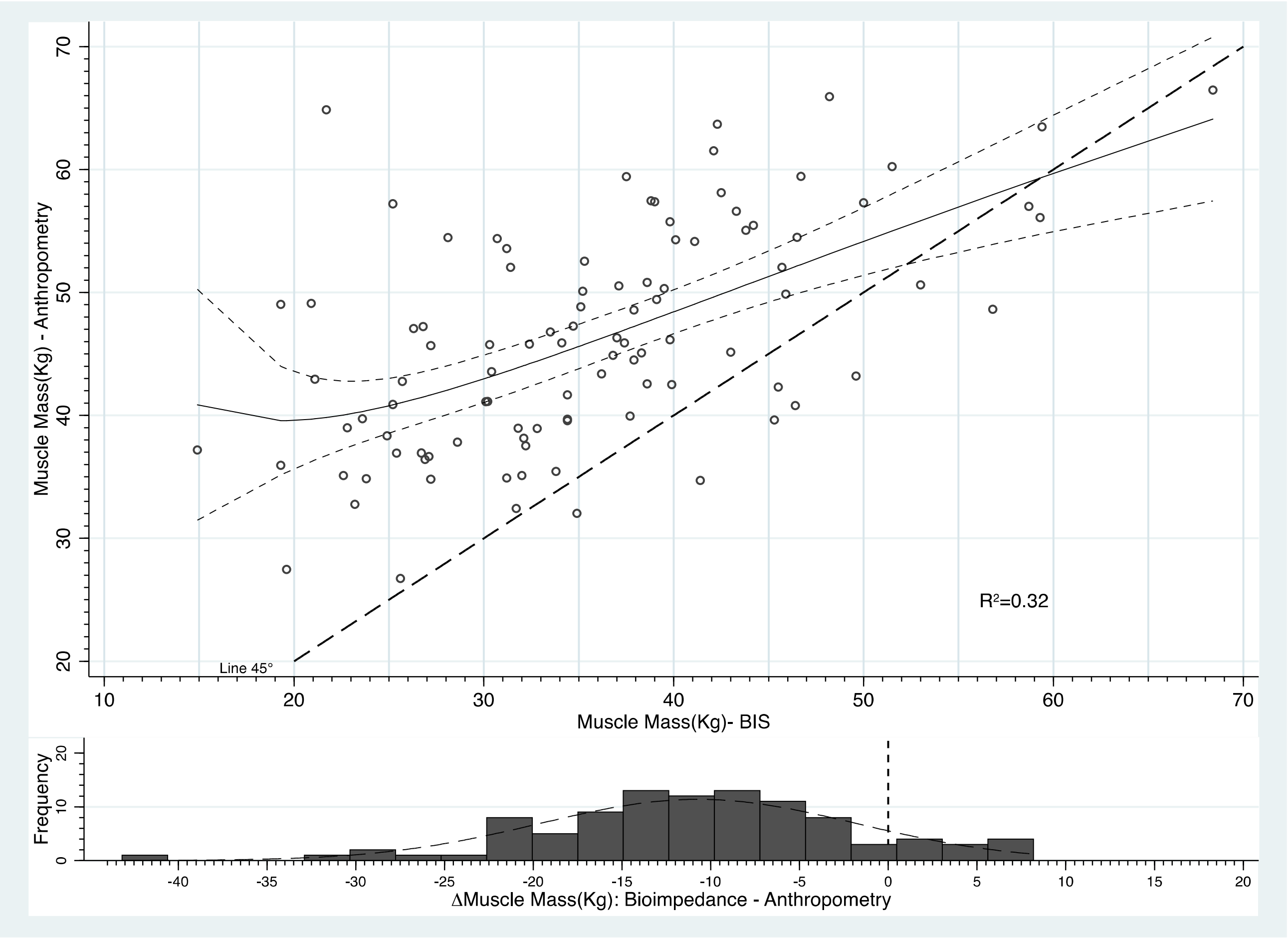

Supplementary Figure 2. Correlation of muscle mass (kg) between anthropometry and BIS in the subgroup of patients with BMI<30.

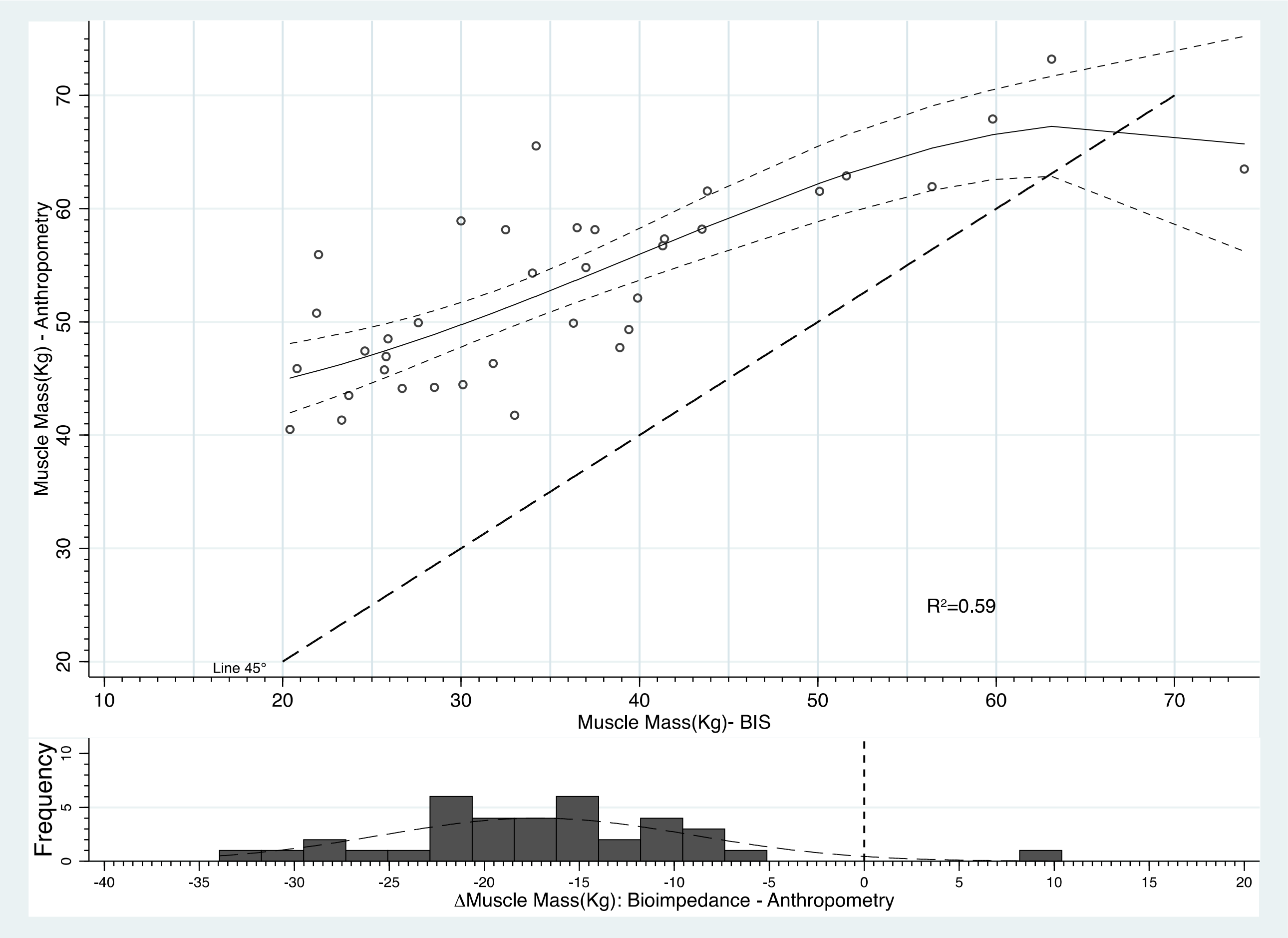

Supplementary Figure 3. Correlation of muscle mass (kg) between anthropometry and BIS in the subgroup of patients with BMI>=30

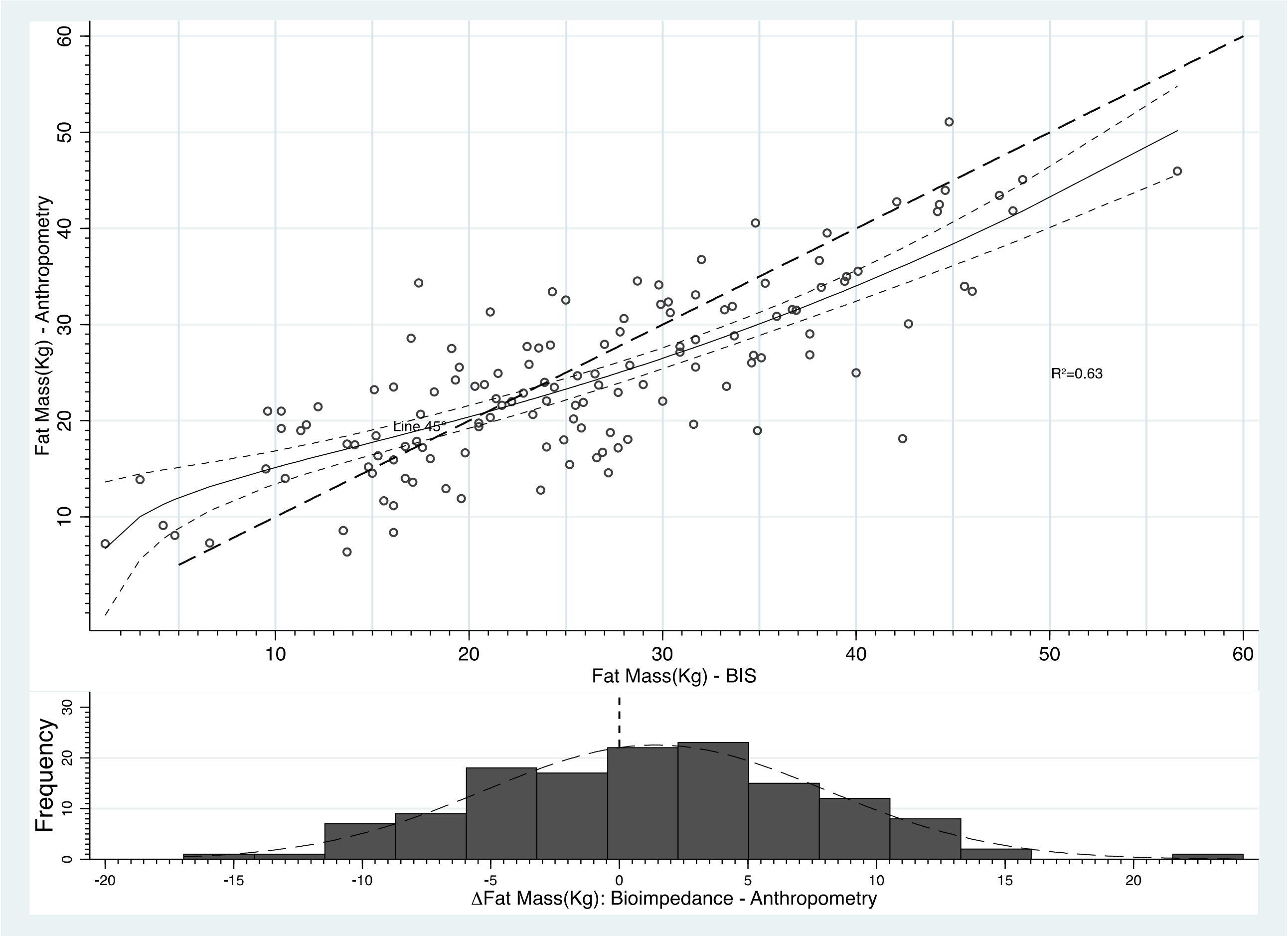

Supplementary Figure 4. Correlation of fat mass (kg) between anthropometry and BIS in the study population

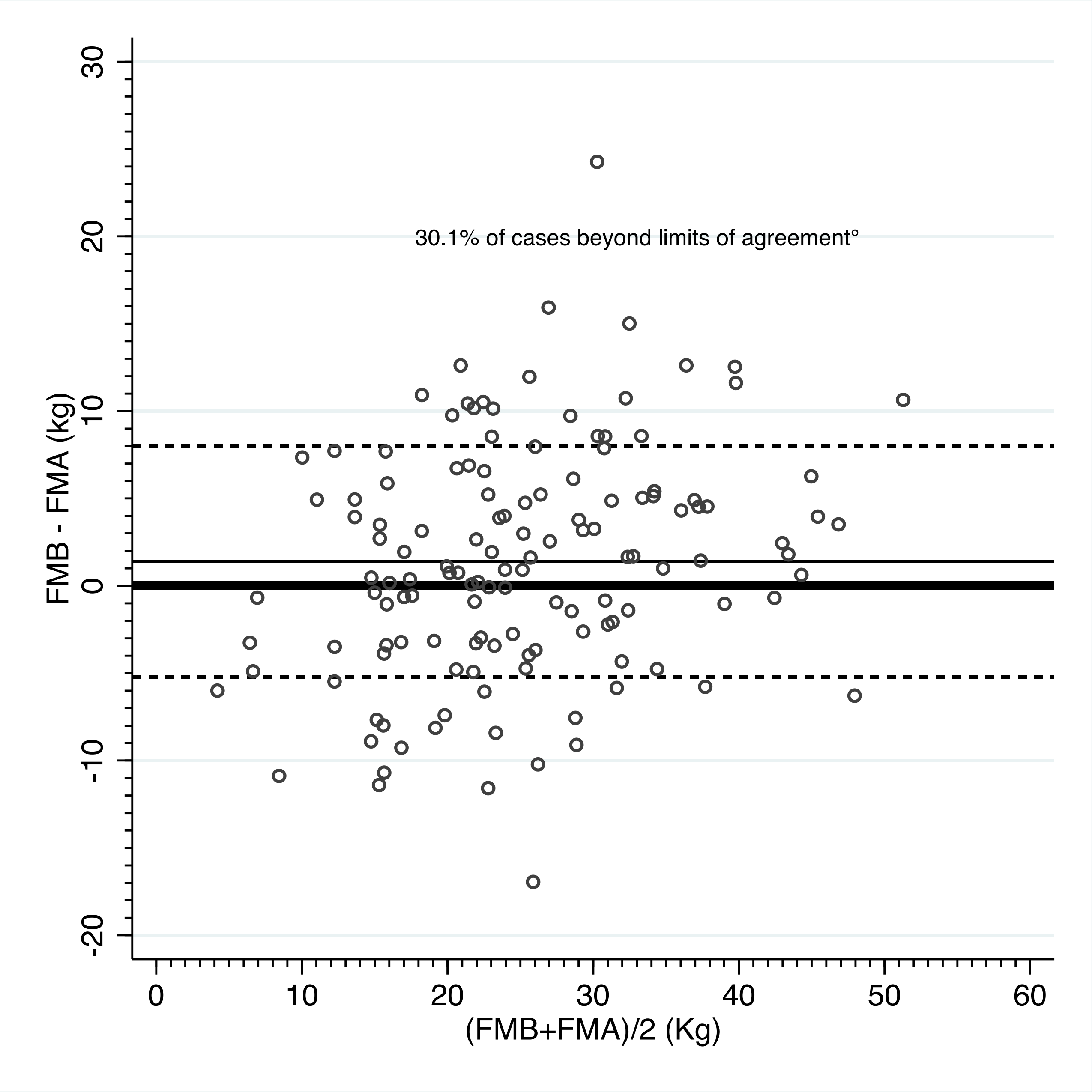

Supplementary Figure 5. Bland-Altmann curve to evaluate the agreement between BIS and anthropometry for the measurement of fat mass
